# Expression of miRNAs in plasma exosomes derived from patients with atrial fibrillation

**DOI:** 10.1002/clc.23461

**Published:** 2020-09-17

**Authors:** Zhang Wei, Zhang Bing, Qian Shaohuan, Wang Yanran, Sun Shuo, Tang Bi, Zhu Feiyu, Zhang Heng, Gao Qin, Kang Pinfang

**Affiliations:** ^1^ Department of Cardiovascular Medicine The First Affiliated Hospital of Bengbu Medical College Bengbu China; ^2^ School of Clinical Medicine Bengbu Medical College Bengbu China; ^3^ Department of Physiology Bengbu Medical College Bengbu China; ^4^ Key Laboratory of Cardiovascular and Cerebrovascular Diseases, PR China Bengbu Medical College Bengbu China

**Keywords:** atrial fibrillation (AF), exosomes, high‐throughput sequencing, miRNA

## Abstract

**Background:**

Studies have revealed the association between exosomes and cardiovascular diseases. However, the typical changes of plasma miRNAs in patients with atrial fibrillation (AF) are still controversial, the use of exosomal miRNAs to diagnose and predict the prognosis of AF has not been described.

**Hypothesis:**

We hypothesized that there were differences in the exosomal miRNAs between AF and normal sinus rhythm (SR) patients, which might be used as the novel biomarkers to reflect the progression of AF.

**Methods:**

miRNAs were isolated from the plasma of patients, and the target genes of differential miRNAs via enrichment analysis to discover potential pathogenesis related to AF. Combined with high‐throughput sequencing results, real‐time PCR was used to verify the relative expression of target miRNAs in patients.

**Results:**

This study confirmed that the expression of plasma‐derived exosomal miRNAs between patients with AF and SR were different. Target gene enrichment analysis suggested that the target genes of 20 miRNAs, which were significantly upregulated were mainly enriched in biological processes such as gene expression process, inflammation response, enzyme modification, etc. Meanwhile, mitogen‐activated protein kinase (MAPK), mammalian target of rapamycin (mTOR), and other pathways were highly enriched. The expressions of miR‐92b‐3p, miR‐1306‐5p, and miR‐let‐7b‐3p had differences between patients with AF and SR.

**Conclusion:**

These miRNAs and target genes were involved in the process of AF through affecting biological processes such as energy metabolism, lipid metabolism, inflammation, and enzyme activity. It suggested that the exosomal miRNAs might be used as the novel biomarkers to reflect the progression of AF.

## 1 INTRODUCTION

Atrial fibrillation (AF), the most common arrhythmia, contributes substantially to cardiac morbidity and mortality in China and the West.^1^ In patients with or without cardiovascular conditions, AF is induced by triggers in the pulmonary vein.^2^ From the etiological point, AF is a manifestation of complex aberrancies in multiple levels, including molecular, cellular, electrical, and structural alterations.^3^, ^4^, ^5^ Furthermore, various risk factors may cause AF, such as heart failure, hypertension, and mitral valve disease.^6^

Exosomes are a type of endogenous extracellular vesicles that have received considerable attention in recent years. They contain bioactive substances, such as regulatory proteins, lipids, DNAs, mRNAs and miRNA‐derived from cells, and their outer surface is rich in CD9, CD63, and other transmembrane proteins which can be used for detection of exosomes.^5^, ^6^, ^7^ As one of the key substances in exosomes, miRNAs can regulate mRNA expression in living organisms, thereby being capable of further regulating various biological pathways in living organisms,^8^ multiple studies have also shown that miRNA can regulate the expression of nearly half of the genes in vivo.^9^, ^10^, ^11^ The study indicated that increased expression levels of exosomal miR‐1306‐5p in patients with heart failure, which were proportional to the expression levels of NT‐proBNP, meanwhile, the expression levels of miR‐1306‐5p can serve as a diagnostic indicator for predicting long‐term prognosis of heart failure.^12^, ^13^ Chen et al discovered that the expression of miR‐320a was increased significantly in patients with coronary heart disease, and meanwhile experiments on rats indicated and confirmed that miR‐320a could promote the expression of inflammatory factors such as IL‐6, MCP‐1, and TNF‐α and further facilitate the formation of atherosclerosis.^14^

While existing studies have revealed the close association between exosomes and cardiovascular diseases, the correlation between exosomes and AF still remains unclear. In the present study, we aimed to identify the difference in exosomal miRNA using high‐throughput sequencing according to the progress of AF, and further explore and analyze the pathogenesis of AF, and at the same time, validate the relative expression of miR‐92b‐3p, miR‐1306‐5p, and miR‐let‐7b‐3p in patients with AF and sinus rhythm (SR) using real‐time PCR technique, based on the results of high‐throughput sequencing.

## 2 MATERIALS AND METHODS

Patients who were admitted to our hospital from June 2018 to May 2019 were primarily enrolled in the study, and later underwent a series of related examinations, including electrocardiogram, cardiac color Doppler ultrasound imaging, and routine blood and biochemical tests. Among them, 23 patients with SR were further selected and included in the SR group; another 26 patients with AF, all of whom underwent radiofrequency ablation in our hospital for AF treatment, were further selected and divided into two groups: Paroxysmal AF group (PaAF group, 13 patients) and Persistent AF group (PeAF group, 13 patients). When patients with AF collect their peripheral blood, all patients with persistent AF have AF rhythm, and all AF is SR. Exosomes were isolated from peripheral venous plasma of all patients for further detection, and three patients were randomly selected from each of the above three groups for subsequent high‐throughput sequencing of exosomal miRNAs, enrichment analysis of target genes and other analytical processes to further explore the biological mechanisms of AF. miRNAs of higher significance were then selected based on the results of high‐throughput sequencing, and real‐time PCR technique was employed to validate the miRNAs in the remaining patients. Ethical approval was given by the medical ethics committee of Bengbu Medical College with the following reference number: 2019KY023.

### 2.1 Inclusion and exclusion criteria

Inclusion Criteria: All enrolled patients first underwent conventional 12‐lead ECG or 24‐hour dynamic electrocardiogram, among whom patients with either AF or SR were then selected based on their electrocardiograms, after detailed inquiry of medical history of then‐selected patients with AF, patients with PaAF and PeAF that met the criteria were further selected,all selected patients were aged 18 to 65 years and agreed to participate in this experiment on a voluntary basis.^15^, ^16^

Exclusion Criteria: This study excluded patients with various types of secondary AF, including AF caused by severe rheumatic heart disease, hyperthyroid‐induced cardiopathy, and congenital heart disease, complicated by severe heart failure, hepatic and renal insufficiency, and malignant tumors, or with severe body infections, systemic autoimmune rheumatic diseases or chronic inflammatory diseases that might affect the interpretation of relevant indicators, or with recent traumatic injuries or surgical trauma, severe physical and mental illnesses and other diseases.^15^, ^16^

### 2.2 Instruments and reagents

Main instruments include: Malvern Nanosight (Malvern Instruments Ltd.,Worcestershire), Real‐Time PCR Instrument (Applied Biosystems), Illumina‐HiSeq4000 Sequencing Platform (Illumina, Inc.), Agilent 2100 Bioanalyzer (Agilent RNA 6000 Nano Kit), etc, main reagents include: Total Exosome Isolation (by Thermo Fisher Scientific Co., Ltd. Shanghai), ACTIN (Servicebio), TRIzol Reagent (Invitrogen CorP. Beijing), Reverse Transcription Kit (Thermo Fisher(China) Scientific Inc. Shanghai), Stem‐loop miRNA 1st Strand Synthesis Kit (Nanjing Vazyme Biotech Co., Ltd.), etc.

### 2.3 Isolation of exosomes and miRNAs

The EDTA anticoagulation tube was used to draw 10 mL of peripheral venous blood in the morning of the next morning after fasting. The plasma was separated by high‐speed centrifugation and stored at −80°C in the refrigerator. At the same time, western blotting was used to detect the concentration of marker proteins on the surface of exosomes, so as to identify the extracted exosomes. Total RNAs were isolated from the exosomes in nine patients using the Trizol method, and the concentration and mass of the samples were determined using the Agilent 2100 Bioanalyzer, rRNAs were depleted using the Epicenter Ribo‐Zero kits, and then following the fragmentation of RNAs, first‐strand cDNAs were further synthesized from these RNAs via reverse transcription, a RNA gene library was constructed using PCR technique, and miRNAs were isolated and purified by high‐resolution polyacrylamide gel electrophoresis (PEAG) to form a miRNA library, which was then sequenced using the Illumina‐HiSeq4000 Sequencing Platform.

### 2.4 Prediction and enrichment analysis of miRNA target genes

The data on exosomal miRNAs obtained from the three groups were compared, and the expression levels of isolated miRNAs were normalized to the default 90th percentile. According to relevant criteria, miRNAs with their |log2 (Flod change)| ≥ 1 and Q value ≤ 0.001 are considered as differential genes, and a Log2 (Flod change) value greater than 0 indicates upregulated miRNA expression, while a Log 2 value less than 0 means signifies downregulated miRNA expression, the Q value refers to FDR adjusted *P*‐value, and Flod change is the ratio between two gene expression levels,^17^, ^18^ a heat map for cluster analysis was created based on the relevant |log2| values, featuring miRNAs with remarkable differences, for further observation of differences in miRNAs among the three groups, meanwhile, prediction of target genes of the selected differential miRNAs was conducted based on RNAhybrid (http://bibiserv.techfak.uni-bielefeld.de/rnahybrid/), miRand (http://www.microrna.org/Microrna/home.do) and TargetScan (http://www.The targetscan.org/) databases, and the gene ontology (GO) and KEGG enrichment analyses of the obtained target genes were also performed.

### 2.5 Validation of miRNA using real‐time PCR

Based on the results of high‐throughput sequencing, miRNA‐1306‐5p was chosen as the indicator for further verification in this experiment. The levels of relative expression of miR‐92b‐3p, miR‐1306‐5p, miR‐let‐7b‐3p in the plasma‐derived exosomes of patients in the verification group were determined using real‐time PCR. PCR primers were as follows, miR‐1306‐5p forward: 5′‐GACCACCTCCCCTGCAA‐3, reverse: 5′‐CAGTGCGTGTCGTGGAGT‐3′, miR‐let‐7b‐3p forward: 5′‐GAAACCACACAACCTACTA‐3′,reverse: 5′‐GGTGAGGTAGTAGGTTGTGTGG‐3′, miR‐4433b‐3p, forward: 5′‐CAGGAGTGGGGGGTGGG‐3′,reverse: 5′‐GCAGGGTCCGAGGTATTC‐3′.

### 2.6 Statistical analysis

Data analysis was conducted using the SPSS25.0 software. Both *t*‐test and chi‐square test were performed for further statistical analysis of all measurement data conforming to a normal distribution. In all statistical results, a difference is deemed statistically significant when the *P*‐value is <.05.

## 3 RESULTS

### 3.1 Comparison of clinical data

Among the nine patients selected for high‐throughput sequencing of plasma samples, the *P*‐value was <.05 for low‐density lipoprotein (LDL) and anteroposterior diameter of left atrium (LAAp), signaling statistically significant differences, Among the 20 patients selected for validation analysis by qPCR, the *P*‐value was <.05 for triglyceride (TG), LA, and left ventricular ejection fraction (LVEF), signifying statistically significant differences. Such results indicated that AF may affect the heart function of patients and change their cardiac structure. See Table 1 below for relevant data.

**TABLE 1:** Comparison of clinical data of each group of samples

	High‐throughput sequencing group					qPCR validation group			
	SR(n = 3)	PaAF(n = 3)	PeAF(n = 3)	F/X^2^	*P*	SR(n = 20)	AF(n = 20)	F/X2	*P*值
Age(Y)	65.33 ± 7.02	61.33 ± 7.77	59 ± 8.72	0.50	.63	58.30 ± 10.36	55.25 ± 8.52	3	.09
Male	1	1	2	0.9	.64	12	10	0.40	.51
BMI(kg/m^2^)	27.37 ± 2.22	29.20 ± 2.59	28.67 ± 2.74	0.42	.68	26.91 ± 2.76	28.91 ± 2.65	0.61	.44
Hypertension	0	1	0	2.25	.33	5	6	0.13	.72
Stroke	1	0	0	2.25	.33	4	3	0.17	.68
Blood biochemical parameters									
WBC10^9^/L	5 ± 1.46	5.91 ± 0.84	6.87 ± 1.29	1.76	.25	6.02 ± 2	6.85 ± 2.76	1.13	.29
PLT 10^9^/L	186.67 ± 10.07	171.33 ± 16.80	185 ± 17.09	0.94	.44	215.2 ± 46.30	220.30 ± 54.59	0.32	.58
D‐di ug/ml	0.25 ± 0.07	0.22 ± 0.46	0.14 ± 0.31	3.06	.12	0.32 ± 0.26	0.24 ± 0.18	2.20	.15
ALT U/L	23 ± 6.56	13.67 ± 2.52	15 ± 5.57	2.86	.13	27.35 ± 15.04	31.85 ± 23.30	2.40	.13
Scr umol/L	52 ± 13.75	63.67 ± 11.37	76 ± 5.29	3.74	.08	63.90 ± 15.33	71.10 ± 19.78	0.90	.35
UA umol/L	397.76 ± 118.97	339 ± 123.60	407 ± 181.43	0.20	.83	361.10 ± 81.14	350.95 ± 82.61	0.20	.66
TG umol/L	1.94 ± 0.71	2.34 ± 1.71	3.41 ± 1.32	1.01	.42	1.62 ± 0.40	2.28 ± 0.79	9.72	<.01
LDLmmol/L	1.50 ± 0.66	2.51 ± 0.28	2.95 ± 0.58	5.89	.04	2.02 ± 0.60	2.21 ± 0.79	1.61	.21
CRPmmol/L	1.47 ± 0.91	1.23 ± 0.64	1.07 ± 0.06	0.29	.75	1.55 ± 2.81	2.06 ± 1.31	0.74	.46
Cardiac ultrasound									
LAAp mm	34.67 ± 2.52	38.33 ± 4.16	45.33 ± 3.51	7.34	.02	31.10 ± 2.85	36.40 ± 5.64	5.34	.03
IVSd mm	7.67 ± 0.58	8.67 ± 0.58	9.00 ± 1.00	2.60	.15	8.2 ± 1.01	8.40 ± 0.88	0.58	.45
LVEF%	62.67 ± 4.04	53.00 ± 2.00	57.00 ± 8.38	1.12	.39	60.20 ± 3.14	55.00 ± 6.37	6.15	.02

Abbreviations: AF, atrial fibrillation; LAAp, anteroposterior diameter of left atrium; LDL, low‐density lipoprotein; LVEF, left ventricular ejection fraction; PaAF, paroxysmal AF group; PeAF, persistent AF group; SR, sinus rhythm; TG, triglyceride.

### 3.2 Test results of exosomal surface protein markers and detection of miRNAs

**FIGURE 1:**
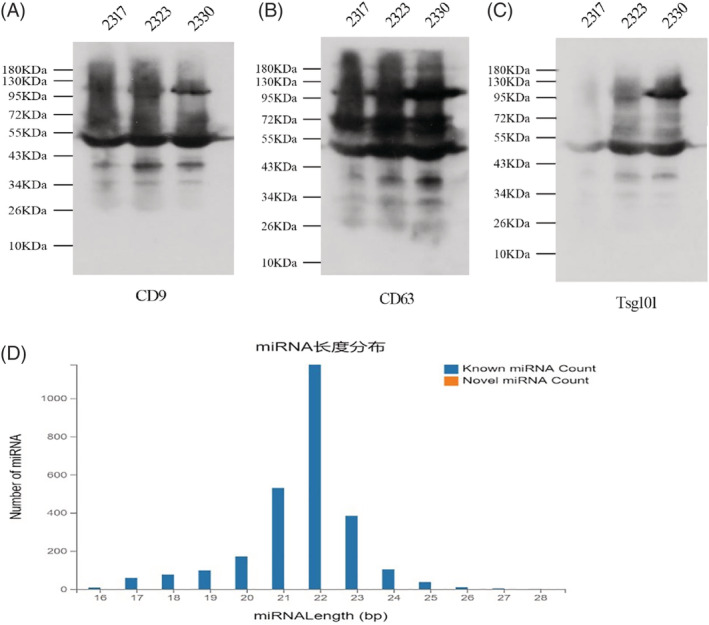
A‐C, Western blotting results of exosomal marker proteins in each group. D, The picture of miRNA length distribution 2317, 2323, and 2330 represent randomly selected samples from the SR, PaAF, and PeAF groups, respectively. PaAF, paroxysmal AF group; PeAF, persistent AF group; SR, sinus rhythm

Exosomal surface protein markers CD9, CD63, and TSG101 detection was conducted using the Western blotting technique. The protein bands of CD9, CD63, and TSG101 were clearly observed among patients in the three groups, indicating that the isolated substances in this experiment were exosomes (Figure 1A‐C). On the other side, the known lengths of miRNAs in human body mostly fall within a range of 20 to 25 nt. Based on this principle, detection of the isolated miRNAs was conducted, and a miRNA length distribution chart was created accordingly to visually evaluate the purity of the isolated miRNAs. The results indicated that the lengths of the extracted miRNAs were mostly centered at around 22 nt, suggesting that the isolated noncoding RNAs in this experiment were miRNAs (Figure 1D).

### 3.3 Statistics of differential genes

**FIGURE 2:**
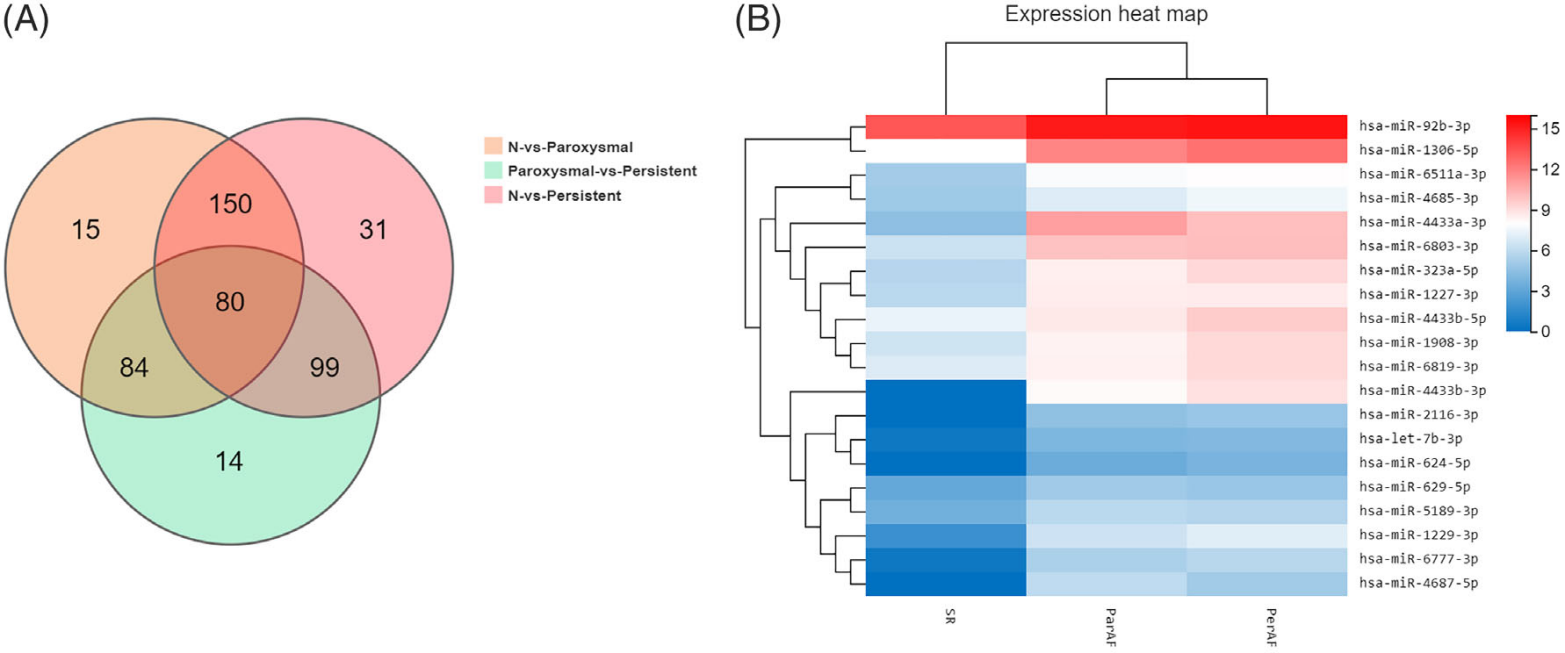
A, Wayne diagram of differential genes in each comparison group. B, Heat analysis of cluster analysis of differentially expressed miRNAs

Differential expression analysis was performed on miRNAs expressed in patients in all the three groups, and a log2 value greater than 0 was set as an indicator of upregulated expression, while a log2 value less than 0 served as an indicator of downregulated expression. The Venn diagram of differential genes created to visually compare the difference in expression between different groups clearly indicated a total of 230 differential genes between the SR vs PaAF groups and the SR vs PeAF groups, of which 80 differential genes were in the PaAF vs PeAF groups(Figure 2A). Through comparison of the differential genes between the above three groups, we subtracted the differential genes in the PaAF vs PeAF groups from the 230 genes between the SR vs PaAF groups and the SR vs PeAF groups, and thus selected a total of 150 differential genes. Among them, 33 were upregulated and 117 downregulated. Under the premise that the Q value was <0.001, the top 20 miRNAs with the highest |log2 (Flod change)| values were further selected. Meanwhile, a heat map/cluster analysis of the selected 20 differential genes was conducted. As shown in Figure 2B, red indicates higher expression levels while blue indicates lower expression levels.

### 3.4 Results of prediction and enrichment analysis of miRNA target genes

Prediction of the target genes of the top 20 markedly upregulated miRNAs listed in the above table was conducted, and enrichment analysis of the isolated target genes was also performed using the KEGG‐pathway and GO databases. In this study, the Q value of <0.001 is regarded as an indicator of significant enrichment, the larger the Rich Ratio value, the higher the degree of enrichment is also in this experiment, pathways were selected based on the Q value and were further enumerated in the form of a graph.

#### 3.4.1 Results of KEGG‐pathway classification and enrichment analysis

**FIGURE 3:**
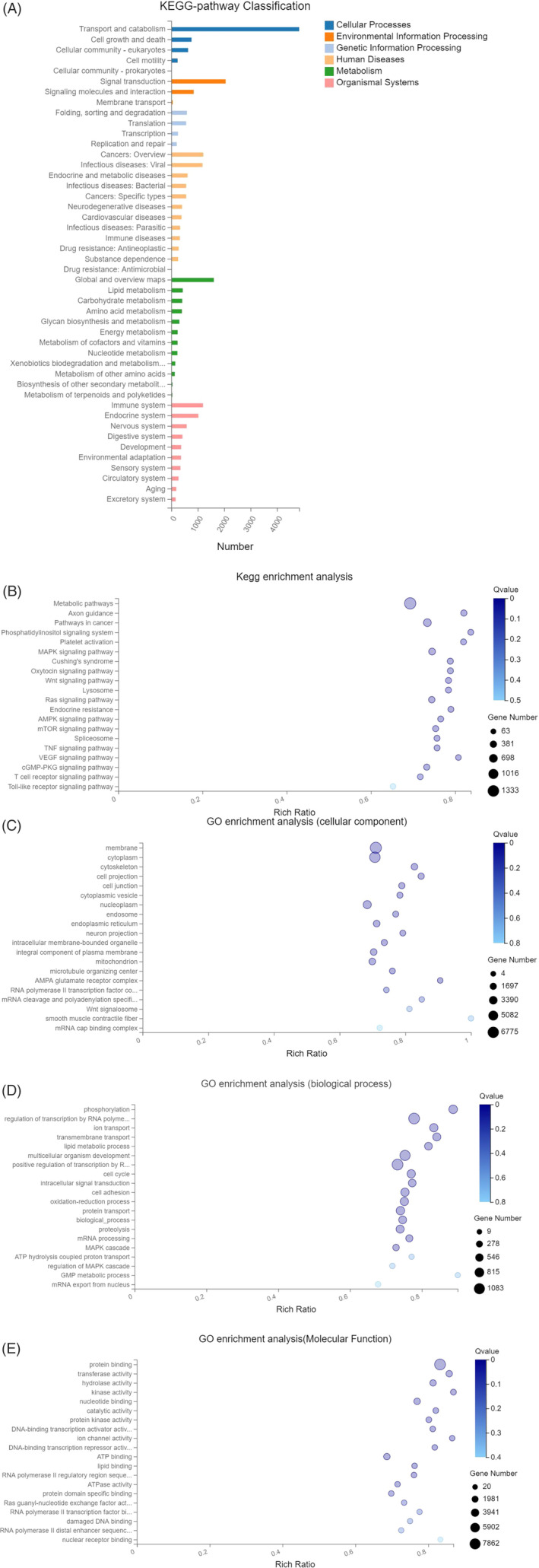
A, Target gene KEGG‐pathway classification chart. B, Enrichment bubble chart. C‐E, Bubble chart of GO enrichment analysis. GO, gene ontology

The results of KEGG‐pathway classification suggested that a large proportion of target genes were enriched in multiple pathways such as transport and catabolism and signal transduction, and the target genes mostly acted on the immune system and endocrine system (Figure 3A). KEGG‐pathway enrichment analysis indicated that highly enriched pathways were as follows: Metabolic pathways (KEGG‐pathway has 1100), mitogen‐activated protein kinase (MAPK) signaling pathway (KEGG‐pathway has4010), Protein processing in endoplamic reticulum (KEGG‐pathway has 4141), Peroxisome (KEGG‐pathway has 4146), cAMP signaling pathway (KEGG‐pathway has 4024), etc., further suggesting that target genes were highly enriched in pathways of energy metabolism, functional transport, inflammatory response, information transmission, etc. See Figure 3A,B for further details.

#### 3.4.2 Results of GO enrichment analysis

Functional enrichment analysis of all target was conducted using the GO database, GO is divided into three parts: Molecular Function, biological process, and cellular component. And indicated that highly enriched pathways were as follows: regulation of transcription by RNA polymerase II (GO: 0006357), phosphorylation (GO: 0016310), transmembrane transport (GO: 0055085), Protein binding (GO: 0005515), oxidoreductase activity (GO: 0016491), nuclear acid binding (GO: 0003676), etc., further suggesting that target genes played a significant role mostly in cytoplasmic and nuclear metabolic process, inflammatory response, transcription, etc. See (Figure 3C‐E) for further details.

### 3.5 Validation of differences in miRNA expression via qPCR

**FIGURE 4:**
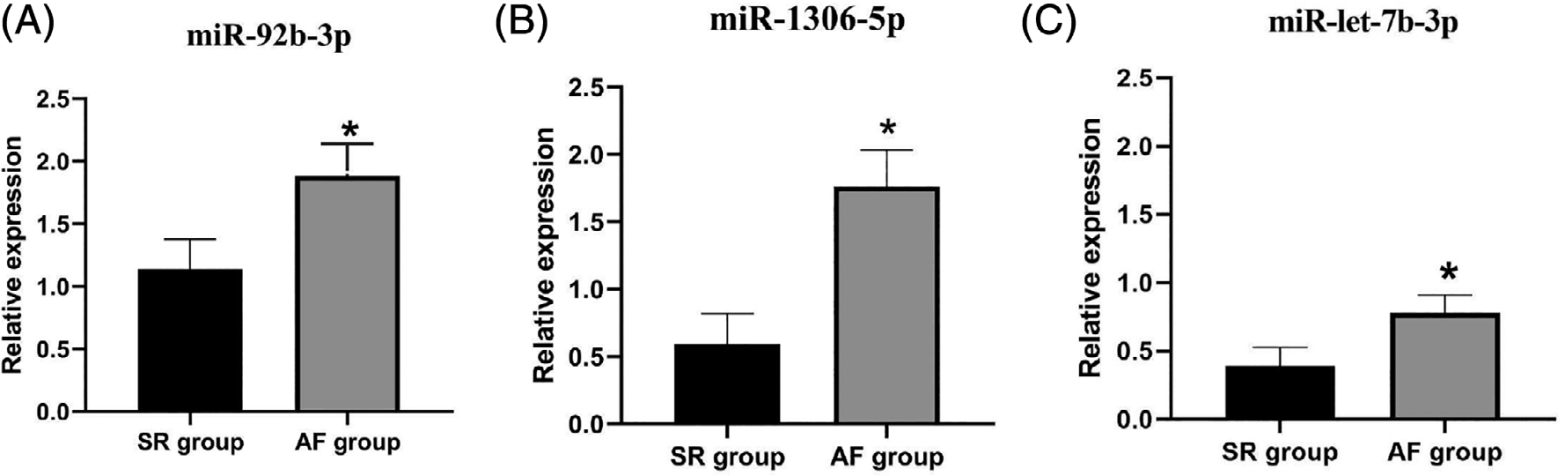
The relative expression levels of serum‐derived exosomal miR‐92b‐3p, miR‐1306‐5p, miR‐let‐7b‐3p in patients. AF, atrial fibrillation; SR, sinus rhythm.*P < .005 vs SR group

The miR‐92b‐3p, miR‐1306‐5p, miR‐let‐7b‐3p was selected from all the upregulated miRNAs for further PCR validation process, based on the results of high‐throughput sequencing. The validation group consisted of 20 SR and AF patients, and all related clinical data were illustrated in Table 1. The expression levels of miRNAs in SR and AF groups were as follows: miR‐92b‐3p (SR group: 1.138 ± 0.240, AF group: 2.398 ± 0.257, AF group: 1.884 ± 0.257, *P* < .005), miR‐1306‐5p(SR group: 0.591 ± 0.228, AF group: 1.762 ± 0.271, *P* < .005), miR‐let‐7b‐3p(SR group: 0.390 ± 0.139, AF group: 1.762 ± 0.130, *P* < .005). The results show that there is a significant difference in the expression levels of miR‐92b‐3p, miR‐1306‐5p, and miR‐let‐7b‐3p in serum exosomes of patients with AF and SR, the results of miRNA verification for these two groups were consistent with those of high‐throughput sequencing. See Figure 4 for further details.

## 4 DISCUSSION

AF is a common type of clinical arrhythmias, long‐lasting presence of AF can lead to other symptoms in patients, including myocardial fibrosis, changes in local myocardial microenvironment, and concurrent inflammatory reactions, which may further aggravate cardiac structural changes and affect the heart function of patients.^19^, ^20^ While existing studies have revealed the close association between exosomes and cardiovascular diseases, the correlation between exosomes and AF still remains unclear. Therefore, this experiment explored the biological pathways in which miRNAs in exosomes may participate in the mechanism of AF, and further to explore and analyze the pathogenesis of AF using high‐throughput sequencing, and at the same time, to validate the relative expression of miR‐92b‐3p, miR‐1306‐5p, and miR‐let‐7b‐3p in patients with AF and SR using real‐time PCR technique based on the results of high‐throughput sequencing.

The miR‐92b‐3p was first proved to be a diagnostic factor for multiple types of cancer, such as rectal and gastric cancer.^21^, ^22^ Later, researchers identified an increase in the expression levels of miR‐92b‐3p after hypoxia or isoflurane stimulated primary cardiomyocytes and fibroblasts in neonatal rats.^23^ Yu et al also found that miR‐92b‐3p could inhibit the expression of HAND2, thereby effectively inhibited Ang‐II‐induced cardiomyocyte hypertrophy in mice.^24^ Hu et al discovered in experiments on rats that miR‐92b‐3p is able to inhibit cardiomyocyte hypertrophy by downregulating the expression of Myocyte‐specific enhancer factor 2D (MEF2D).^25^ Likewise, the correlation between miR‐let‐7b‐3, miR‐1306‐5p and cardiovascular diseases has also been confirmed.^12^, ^13^ In addition, Ham et al found that in rats, let‐7b protected cardiomyocytes against ischemia‐induced injury by directly acting on caspase‐3 signals.^26^ A significant increase in the expression of miR‐let‐7b‐3p in the plasma of patients with myocardial infarction can serve as one of the indicators for diagnosis of myocardial infarction.^27^ In this regard, the correlationamong the three miRNAs (miR‐92b‐3p, miR‐1306‐5p and miR‐let‐7b‐3p) verified in this experiment and cardiovascular diseases has been conformed, but lack of experimental evidence to associate such miRNAs with AF; meanwhile, the PCR results revealed the significant increase in the expressions of all three miRNAs in the serum of patients with AF, indicating the possible role of these miRNAs in the pathogenesis of AF.

On the other hand, the enrichment analysis indicated that the target genes were highly enriched in multiple signaling pathways, including MAPK and mammalian target of rapamycin (mTOR) signaling pathways. MAPK is a group of serine‐threonine protein kinases that can be activated by a variety of stimuli, including cytokines, neurotransmitters, hormones and cell adhesion, and participate in the transmission of various signaling pathways.^28^, ^29^ Zhou et al discovered that the expression of miR‐30‐3p was increased significantly in the serum of patients with coronary heart disease, and that meanwhile, miR‐30‐3p inhibited the inflammatory responses and apoptosis induced by NF‐κB signals and MAPK pathway and further alleviated coronary artery injury.^30^ mTOR can be affected by multiple factors; including extracellular microenvironments, AMP/ATP energy and oxidative stress, and can exert considerable effects on biological processes such as protein regulation and cell proliferation and apoptosis.^31^, ^32^ Existing studies have indicated that mTOR can affect various biological processes, including cardiomyocyte proliferation, cardiac remodeling, and energy metabolism, in patients with cardiovascular diseases.^33^, ^34^ Jin et al also found that miR‐496 was able to remedy hypoxia‐induced cardiomyocyte apoptosis via PI3k/Akt/mTOR signaling pathways. The above demonstration indicated that MAPK and mTOR signaling pathways play an essential role in the progression of cardiovascular diseases, and at the same time, various miRNAs inside human body play significant a role in the progression of cardiovascular diseases via the above signaling pathways.^35^ In addition, AF can lead to a wide range of abnormalities, including changes in the local myocardial microenvironments, inflammatory responses, mitochondrial dysfunction, abnormal lipid metabolism, and further facilitate the initiation and maintenance of AF,^36^, ^37^, ^38^ meanwhile, the enrichment analysis in this experiment has also confirmed the role of miRNAs in the formation of mechanisms of AF via the above mechanisms. Therefore, miRNAs in the serum are able to participate in the formation of mechanisms of AF through various factors, and meanwhile, the results of this enrichment analysis can provide research directions for further exploring the specific mechanisms of miRNAs affecting the initiation of AF.

## 5 CONCLUSION

This experiment confirmed that patients with AF and patients with SR have greater differences in plasma miRNAs expression. These differential miRNAs (such asmiR‐92b‐3p, miR‐1306‐5p, miR‐let‐7b‐3p) may participate in the development of AF by affecting multiple biological pathways and signal channels. These data provide a positive direction for further research on the relationship between exosomal miRNAs and AF and the exploration of new biomarkers of AF, and provide a research foundation for the further treatment of AF.

## CONFLICT OF INTEREST

The authors declare conflict of interest.

## AUTHOR CONTRIBUTIONS

Qin Gao and Pinfang Kang contributed equally to this work.

## ETHICS STATEMENT

Ethical approval was given by the medical ethics committee of Bengbu Medical College with the following reference number: 2019KY023.

## Data Availability

The data used to support the findings of this study are available from the corresponding author upon request.
